# Quantitative determination and validation of 17 cannabinoids in cannabis and hemp using liquid chromatography-tandem mass spectrometry

**DOI:** 10.1007/s00216-020-02862-8

**Published:** 2020-08-24

**Authors:** Garnet McRae, Jeremy E. Melanson

**Affiliations:** National Research Council of Canada, Metrology, 1200 Montreal Road, Ottawa, ON K1A 0R6 Canada

**Keywords:** Cannabinoids, Cannabis, Hemp, Liquid chromatography-mass spectrometry, Validation

## Abstract

The increase in production of cannabis for medical and recreational purposes in recent years has led to a corresponding increase in laboratories performing cannabinoid analysis of cannabis and hemp. We have developed and validated a quantitative liquid chromatography-tandem mass spectrometry (LC-MS/MS) method that is simple, reliable, specific, and accurate for the analysis of 17 cannabinoids in cannabis and hemp. Liquid-solid sample extraction coupled with dilution into a calibration range from 10 to 10,000 ng/mL and LC-MS/MS analysis provides quantification of samples ranging from 0.002 to 200 mg/g (0.0002 to 20.0%) in matrix. Linearity of calibration curves in methanol was demonstrated with regression *r*^2^ ≥ 0.99. Within-batch precision (0.5 to 6.5%) and accuracy (91.4 to 108.0%) and between-batch precision (0.9 to 5.1%) and accuracy (91.5 to 107.5%) were demonstrated for quality control (QC) samples in methanol. Within-batch precision (0.2 to 3.6%) and accuracy (85.4 to 111.6%) and between-batch precision (1.4 to 6.1 %) and accuracy (90.2 to 110.3%) were also evaluated with a candidate cannabis certified reference material (CRM). Repeatability (1.5 to 12.4% RSD) and intermediate precision (2.2 to 12.8% RSD) were demonstrated via analysis of seven cannabis samples with HorRat values ranging from 0.3 to 3.1. The method provides enhanced detection limits coupled with a large quantitative range for 17 cannabinoids in plant material. It is suitable for a wide range of applications including routine analysis for delta-9-tetrahydrocannabinol (Δ^9^-THC), delta-9-tetrahydrocannabinolic acid (Δ^9^-THCA), cannabidiol (CBD), cannabidiolic acid (CBDA), and cannabinol (CBN) as well as more advanced interrogation of samples for both major and minor cannabinoids.

**Electronic supplementary material:**

The online version of this article (10.1007/s00216-020-02862-8) contains supplementary material, which is available to authorized users.

## Introduction

With the legalization of recreational cannabis in Canada and medicinal cannabis in many other jurisdictions, a large number of analytical laboratories servicing the cannabis industry have emerged to address the growing need for cannabis testing. The lack of standardization in cannabis testing has resulted in a wide variety of methods being used, which has undoubtedly contributed to the high variability of results between testing laboratories [1, 2]. However, associations such as AOAC, ASTM, and US Pharmacopeia [3] are working towards developing standardized methods to help resolve this issue.

**Fig. 1:**
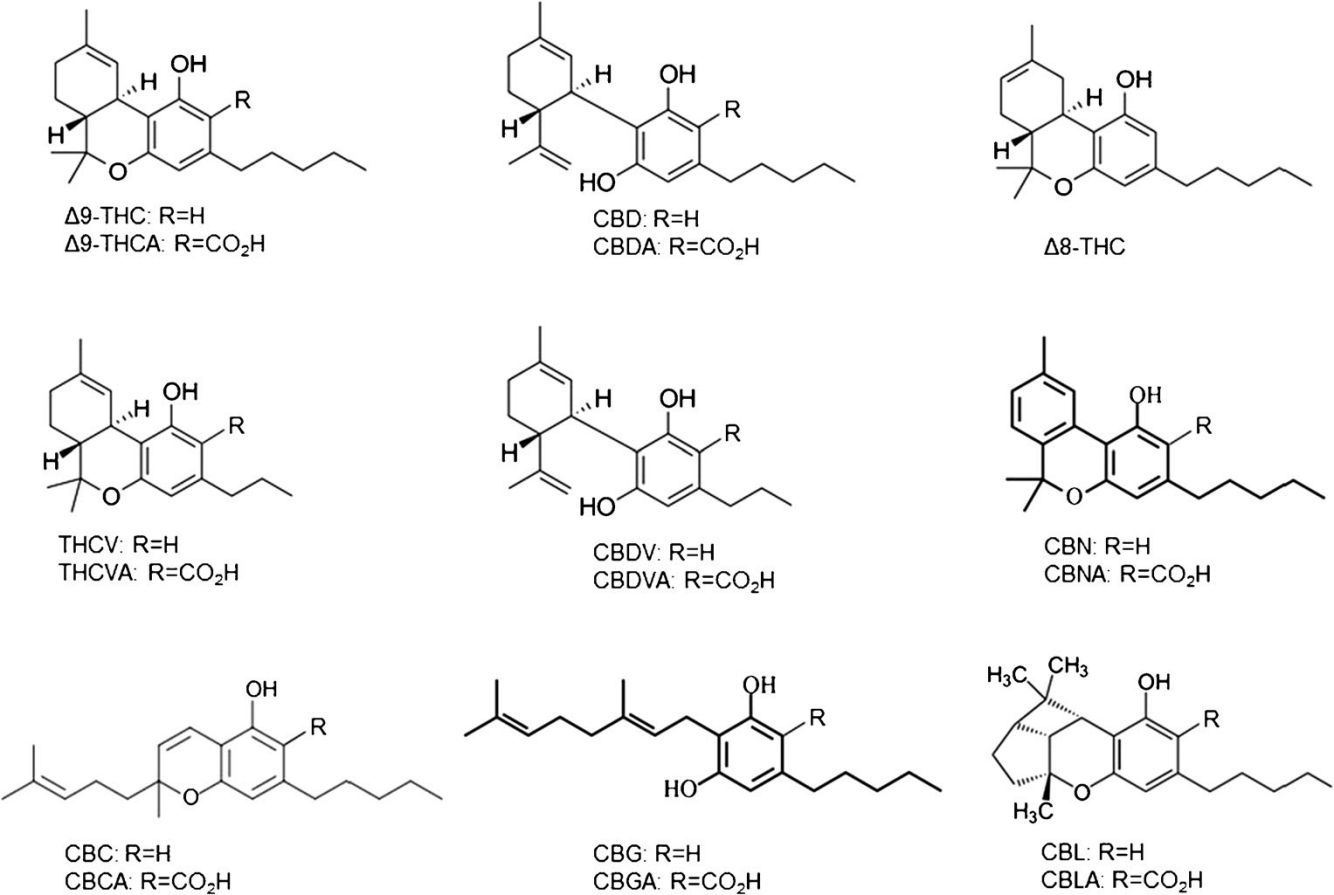
Chemical structures of the 17 cannabinoids targeted in the reported method

Many of the methods currently in use focus primarily on the four major cannabinoids, Δ^9^-THC, Δ^9^-THCA, CBD, CBDA, and CBN, to satisfy testing and labeling requirements for cannabis [4] and to meet the regulatory guidelines for hemp [5]. However, there are many other cannabinoids known to be present in cannabis and hemp for which commercial reference standards are available, including cannabigerol (CBG), cannabigerolic acid (CBGA), cannabinolic acid (CBNA), cannabichromene (CBC), cannabichromenic acid (CBCA), tetrahydrocannabivarin (THCV), tetrahydrocannabivarinic acid (THCVA), cannabidivarin (CBDV), cannabidivarinic acid (CBDVA), cannabicyclol (CBL), cannabicyclolic acid (CBLA), and Δ^8^-tetrahydrocannabinol (Δ^8^-THC) (Fig. 1). Recent reviews of analytical methods for cannabinoids have discussed analytical techniques for the major and minor cannabinoids, revealing few methods providing adequate quantitative analysis of many of the minor cannabinoids [6, 7]. The methods include high-performance liquid chromatography with ultraviolet detection (HPLC-UV) [8–13], liquid chromatography tandem mass spectrometry (LC-MS/MS) [13–18], and nuclear magnetic resonance (^1^H-NMR) spectroscopy [19]. Many of these techniques are limited in sensitivity and specificity, and GC is limited by its inability to directly quantitate the acidic cannabinoids without derivatization [20, 21]. Many methods are quantitative for the major cannabinoids (THC, THCA, CBD, and CBDA) and some of the minor cannabinoids; however, many of the minor neutral and acidic cannabinoids are not quantified due to sensitivity and specificity limitations.

While the large range of cannabinoid concentrations observed in cannabis, and to a lesser extent hemp, is difficult to cover in a single analysis, one approach is to use a wide-range calibration curve coupled with appropriate dilutions of the extracts [14, 18]. Use of a single sample dilution limits the quantifiable range, usually with a sacrifice at the lower concentration ranges. HPLC-UV is commonly used due to the low cost of laboratory set-up and operation [11, 12, 22, 23]. This technique, while providing adequate quantitative results for the major cannabinoids at higher concentration levels, lacks sensitivity and specificity for cannabinoids at lower concentrations [11, 12, 22, 23], limiting the achievable lower limit of quantitation (LLOQ) in matrix. While complete separation is possible for subsets of cannabinoids up to 8 or 12, LC-UV is generally not capable of resolving larger suites of cannabinoids. In some cases, the resolution of challenging cannabinoid pairs relies of precise pH control [11], but this can be problematic and limit the robustness of a method [3]. A fast, 5-min HPLC-DAD method [8] has been reported; however, the LOQ is high at 10 μg/mL, CBDA and most of the minor cannabinoids were not evaluated, and there is no evidence to indicate that many of the cannabinoids do not co-elute.

LC-MS/MS is a sensitive and specific technique which allows the analysis of both major and minor cannabinoids at low LLOQs in the same method [14, 18]. While not yet commonplace for the routine analysis of cannabinoids in plant material, it is the method of choice for the analysis of cannabinoids and metabolites in other complex matrices such as urine, blood, plasma, and oral fluid [24–27]. Application of the LC-MS/MS method presented here achieves low LLOQs for 17 cannabinoids with a range of 0.002 to 200 mg/g in matrix by employing a large calibration range with appropriate sample extraction and dilution. The method has been validated according to AOAC [28] and ASTM [29] guidelines in both cannabis and hemp matrices.

## Experimental

### Materials and reagents

CBD was purchased from Toronto Research Chemicals and certified for purity at NRC using quantitative NMR (qNMR) [30] with NIST SRM 350b benzoic acid as internal standard [31]. Reference standards of the other cannabinoids and isotopically labeled cannabinoids were purchased from Cerilliant (Round Rock, Texas, USA). The neutral cannabinoids included Δ^9^-THC, Δ^8^-THC, CBG, CBN, CBC, THCV, CBDV, and CBL and were provided at 1.0 mg/mL in methanol (CBL provided at 1.0 mg/mL in acetonitrile). The acidic cannabinoids included Δ^9^-THCA, CBDA, CBGA, CBNA, CBCA, THCVA, CBDVA and CBLA and were provided at 1.0 mg/mL in acetonitrile (CBLA provided at 0.5 mg/mL in acetonitrile). The isotopically labeled cannabinoids included Δ^9^-THC-d3, CBD-d3, and CBN-d3, which were provided at 0.1 mg/mL in methanol. Dried cannabis and hemp samples were obtained from licensed producers in Canada via the Ontario Cannabis Store. A candidate NRC certified reference material for cannabis was used for validation and quality control purposes. This material has been rigorously tested to be homogeneous and stable with respect to the 14 cannabinoids it contains, with value assignment for cannabinoids based on a combination of results from a validated LC-UV method [3, 11] and a more targeted version of the LC-MS/MS method reported here that employs narrow calibration ranges that bracket the cannabinoid levels.

Ultrapure water was collected from a Millipore Milli-Q Advantage A10 mixed bed ion exchange system fed with reverse osmosis domestic water (Jaffrey, New Hampshire, USA). Optima® grade acetonitrile, methanol, and formic acid were from Fisher Scientific (Fair Lawn, NJ, USA).

### Solution and calibration curve preparation

Working solutions, each containing all 17 cannabinoids, were volumetrically prepared at 50.0 μg/mL in methanol. The solutions were then further diluted to prepare calibration standards and QC samples as shown in Table S1 (see Electronic Supplementary Material, ESM) using separate working solutions for standards and QC samples. All solutions were stored at − 20 °C. A calibration curve with the same concentration of all 17 cannabinoids for each standard level was prepared instead of a calibration curve prepared with cannabinoids at different concentrations based on typical matrix concentrations [11] (i.e., high concentrations for THC, THCA, CBD, and CBDA and lower concentrations for the other cannabinoids). Keeping the concentrations the same for all cannabinoids limited the introduction of concentration bias due to minor impurities in the reference standards and any potential cannabinoid inter-conversion (i.e., acids to neutrals) and degradation of THC to CBN.

Calibration standards were used to generate calibration curve regressions while QC sample concentrations were derived from the regressions to verify accuracy and precision of the method. The LLOQ (lower limit of quantitation) and ULOQ (upper limit of quantitation) were set to 10 and 10,000 ng/mL respectively for all cannabinoids with the 1000-fold calibration range resulting in a higher likelihood that diluted matrix extract concentrations fell within the calibration curve limits. Matrix sample results were derived from the regressions followed by calculations to account for matrix sample mass, extraction solvent volume, and dilutions with final results reported in mg/g. Calibration curve regressions, QC sample results, and matrix sample results were generated using Xcalibur software, Version 4.0.27.10 (Thermo Scientific, San Jose, CA, USA).

### Sample preparation

Cannabis samples (≥ 2 g of dried flower) were milled to a fine powder via cryogenic grinding. A freezer/mill (SPEX, Metuchen, NJ, USA) was used to cryogrind the materials. A program was set to pre-chill the samples for 2 min, grind for 2 min, and then repeat the process 2 more times, resulting in a finely milled powder. Hemp samples were received as milled materials and were mixed well and extracted directly without further milling.

### Sample extraction

Cannabinoids were extracted from triplicate 100-mg subsamples via a liquid-solid extraction procedure using 5 mL of methanol:water 80:20 [11] by vortexing on a multi-tube vortexer (Troemner, Thorofare, NJ, USA) for 1.5 min. The samples were centrifuged on a Sorvall Legend X1R centrifuge (Thermo Electron, Osterode am Harz, Germany), the supernatant retained, and the procedure repeated 3 times for complete extraction. The combined extracts (20 mL) were mixed well and aliquots were further diluted 1/10 and 1/100 with methanol. All samples, standards, and QC samples were transferred (100 μL) to HPLC vials containing glass inserts and internal standard (50 μL, 500 ng/mL in methanol) was added prior to injection onto the LC-MS system.

### HPLC

An Agilent 1290 Infinity I UPLC system equipped with a binary pump, solvent degasser, column heater, and temperature controlled autosampler (Agilent Technologies, Mississauga, ON, Canada) was used for chromatographic separation. The column was an Ace-3, C18-Amide, 3 μm, 100 × 2.1 mm column with a guard column (Ace-3, C18-Amide, 3 μm, 10 × 2.1 mm) (Advanced Chromatography Technologies, Aberdeen, Scotland) and was controlled at 40 °C. The mobile phases consisted of (A) 100:0.1 water:formic acid and (B) 100:0.1 acetonitrile:formic acid at a flow rate of 0.5 mL/min. The separation was achieved using a gradient as follows: 0–5 min, 57–70% B; 5.0–11.0 min, 70–75% B; 11.0–13.0 min, 75–80% B; 13.0–14.0 min, 80–95% B, followed by a 4.0-min wash at 98% B and column re-equilibration at 57% B for 4.0 min for a 21-min total run time. The solvent flow was diverted to waste from 0 to 4 min, to the mass spectrometer from 4 to 17 min, and to waste from 17 to 21 min. The autosampler was maintained at 4 °C and the injection volume was set to 1 μL.

### ESI-MS/MS

A TSQ Quantiva triple quadrupole mass spectrometer (Thermo Scientific, San Jose, CA, USA), equipped with electrospray ionization (ESI) operating in positive ion mode, was used for cannabinoid analysis. Cannabinoids were individually infused into mobile phase directed to the ESI source of the mass spectrometer via an infusion pump to determine and optimize MS/MS parameters for each cannabinoid. Two different ion transitions were used for all cannabinoids. Ion spray voltage was set to 4000 V, Sheath gas to 50, Aux gas to 20 and Sweep Gas to 2 arbitrary units. The ion transfer tube was set to 325 °C and the vaporizer temperature was set to 280 °C. Argon was used as collision gas at 1.5 mTorr and Q1/Q3 resolution was set to 0.7. The ion transitions and MS voltage parameters are listed in Table 1.

**Table 1:** LC-MS/MS acquisitions parameters for the 17 cannabinoids and internal standards

Name	Q1 (*m/z*)	Q3 (*m/z*)	Q3 (*m/z*)	RF (V)	CE (eV)	CE (eV)	Dwell (ms)	Internal standard	RT (min)
CBDV	*287.2*	*165.1*	123.1	*80*	*23*	30	40	CBN-d3	5.2
THCV	*287.2*	*165.1*	123.1	*80*	*23*	30	40	CBN-d3	6.3
Δ^9^-THC	*315.2*	*193.1*	135.1	*88*	*21*	20	40	THC-d3	9.5
CBD	*315.2*	*193.1*	135.1	*88*	*21*	20	40	CBD-d3	7.7
CBC	*315.2*	*193.1*	135.1	*88*	*21*	20	40	CBN-d3	10.5
Δ^8^-THC	*315.2*	*193.1*	135.1	*88*	*21*	20	40	CBN-d3	10.0
CBG	*317.2*	*193.1*	123.1	*74*	*16*	32	40	CBN-d3	8.9
CBN	*311.2*	*223.1*	241.1	*92*	*22*	18	40	CBN-d3	9.0
CBL	*315.2*	*235.1*	81.1	*77*	*18*	30	40	CBN-d3	11.3
CBDVA	*313.2*	*191.1*	233.1	*100*	*26*	20	40	CBN-d3	7.0
THCVA	*313.2*	*191.1*	233.1	*100*	*26*	20	40	CBN-d3	9.0
Δ^9^-THCA-1	*341.2*	*219.1*	–	*110*	*26*	–	40	THC-d3	13.3
Δ^9^-THCA-2	359.2	–	219.1	85	–	25	40	THC-d3	13.3
CBDA-1	*341.2*	*219.1*	*–*	*110*	*26*	–	40	CBD-d3	10.6
CBDA-2	359.2	–	219.1	85	–	25	40	CBD-d3	10.6
CBCA-1	*359.2*	*219.1*	–	*70*	*27*	–	40	CBN-d3	14.3
CBCA-2	341.1	–	219.1	110	–	25	40	CBN-d3	14.3
CBGA	*343.2*	*219.1*	261.1	*96*	*23*	16	40	CBN-d3	13.6
CBNA	*337.2*	*235.1*	253.1	*140*	*25*	23	40	CBN-d3	12.6
CBLA	*359.2*	*261.1*	219.1	*85*	*25*	32	40	CBN-d3	14.6
THC-d3	*318.2*	*196.1*	135.1	*85*	*21*	20	40	N/A	9.5
CBD-d3	*318.2*	*196.1*	135.1	*85*	*21*	20	40	N/A	7.7
CBN-d3	*314.2*	*223.1*	241.1	*92*	*22*	18	40	N/A	9.0

Italic values indicate quantitation ion parameters and non-italic values indicate confirmation ion parameters. SRM transitions, optimized potentials, dwell times, and retention times of the cannabinoids and internal standards. Q1 (*m/z*) and Q3 (*m/z*) are the mass to charge ratios of the molecular ion selected in Q1 and the fragment ion selected in Q3 respectively. RF (radio frequency) and CE (collision energy) are optimized potentials and Dwell is the dwell time in milliseconds for each ion transition. RT is the chromatographic retention time of each cannabinoid

### Validation

The method validation was based on AOAC [28] and ASTM [29] guidelines. Specifically, the method was validated for specificity, selectivity, recovery, ion suppression, linearity, QC sample precision, accuracy and stability, sample repeatability and intermediate precision, extract stability, and processed sample stability.

## Results and discussion

### LC-MS/MS method development

Each cannabinoid was individually infused into the LC-MS/MS system to determine the molecular ion and product ions and optimize lens voltages. The most sensitive molecular ion→product ion transitions were monitored for quantitation for all cannabinoids with two sets of transitions monitored for THC/CBD (*m/z* 315 → *m/z* 193 and *m/z* 315 → *m/z* 135) and Δ^9^-THCA/CBDA (*m/z* 341 → *m/z* 219 and *m/z* 359 → *m/z* 219). Five neutral cannabinoids, Δ^9^-THC, Δ^8^-THC, CBD, CBC, and CBL, have the same mass and product ions, although CBL showed different product ion response ratios with the *m/z* 235 product ion showing the highest response. The acid cannabinoids were found to readily lose H_2_O in the source, producing both the molecular ion [M + H]^+^ and [M-H_2_O + H]^+^. Both ions were optimized and monitored for Δ^9^-THCA and CBDA. Differences in response and baseline were observed for the two species and this difference was exploited to improve CBCA quantitation, which is more challenging due to its elution after Δ^9^-THCA. The *m/z* 359 → *m/z* 219 transition provided approximately the same CBCA response as the *m/z* 341 → *m/z* 219 transition; however, the Δ^9^-THCA response for the *m/z* 359 → *m/z* 219 transition was approximately 4× lower than for the *m/z* 341 → *m/z* 219 transition. This effect enhances the ability to quantitate low concentrations of CBCA in the presence of high concentrations of THCA. It should be noted that while negative ion mode was evaluated for the acidic cannabinoids, it generated greater variability in peak areas and overall reduced robustness of the method and therefore was not pursued further.

**Fig. 2:**
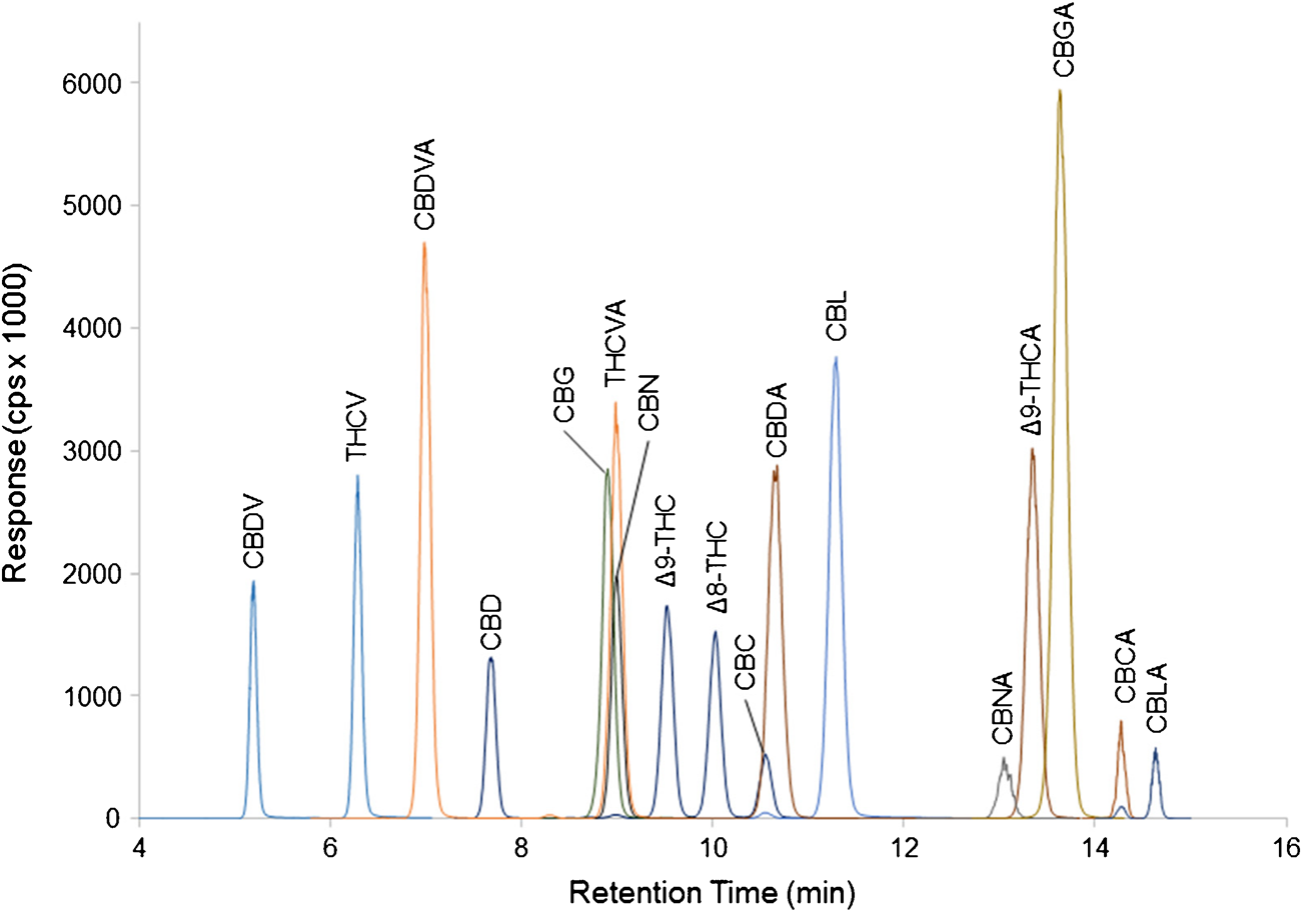
LC-MS/MS chromatogram of 17 cannabinoids in a calibration standard at a concentration of 1000 ng/mL for each cannabinoid

Chromatography was developed using mobile phases consisting of water and acetonitrile with 0.1% formic acid and a C18-amide column. This column provided a unique retention with each acid eluting later than its corresponding neutral (Fig. 2). A stepped gradient was used to optimize the separation of cannabinoids within ± 2 *m/z.* CBN, CBG, and THCVA co-eluted as well as CBC and CBDA; however, these cannabinoids did not interfere with each other due to their unique mass and/or product ions. Note that while a C18-amide column was employed, other conventional C18 columns could easily be used as alternatives using an appropriate gradient.

**Fig. 3:**
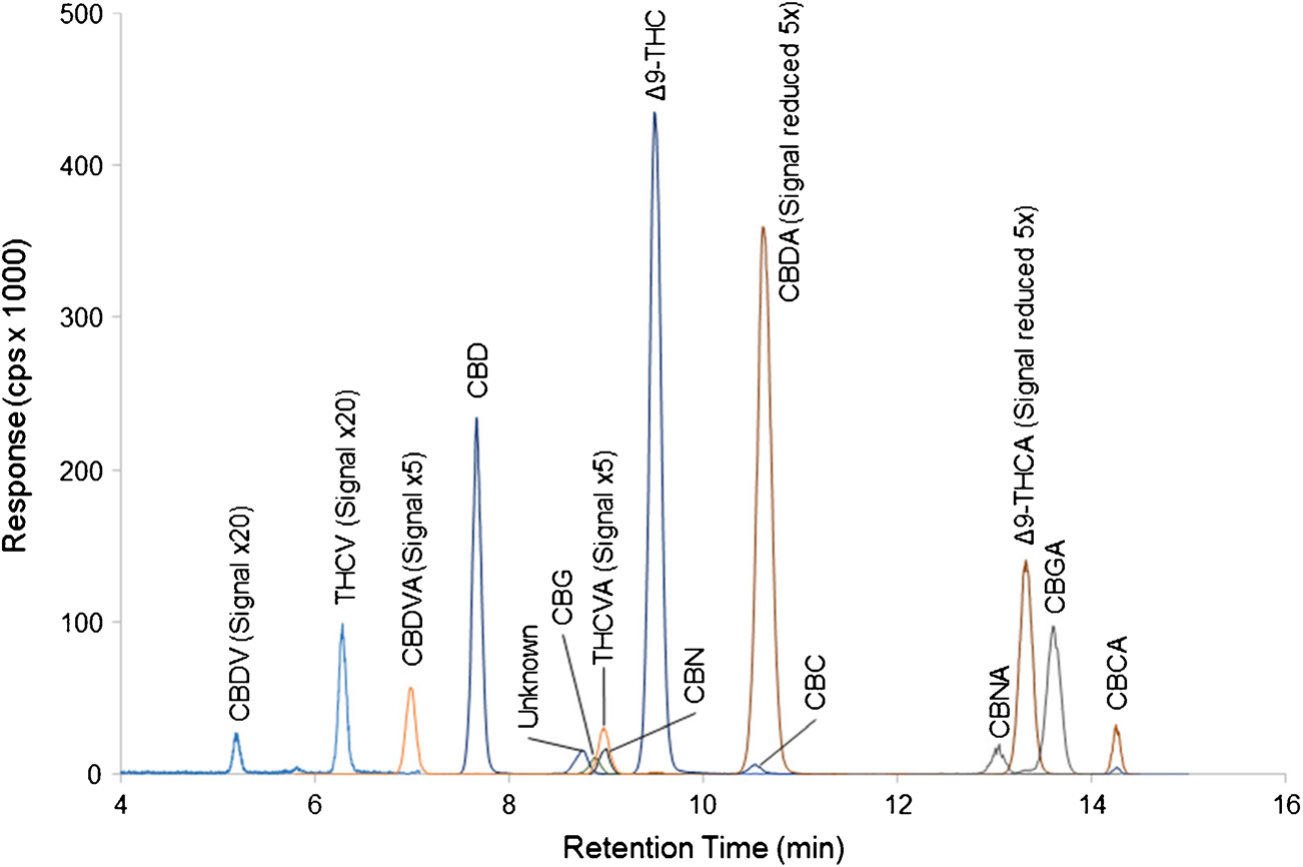
LC-MS/MS chromatogram of 17 cannabinoids in a cannabis sample extracted with methanol:water and diluted 1/100 with methanol. The responses of minor cannabinoids CBDV, CBDVA, THCV, and THCVA have been enhanced 5- to 20-fold while the responses of CBDA and Δ^9^-THCA have been reduced 5-fold to allow visualization of all peaks in the chromatogram

**Fig. 4:**
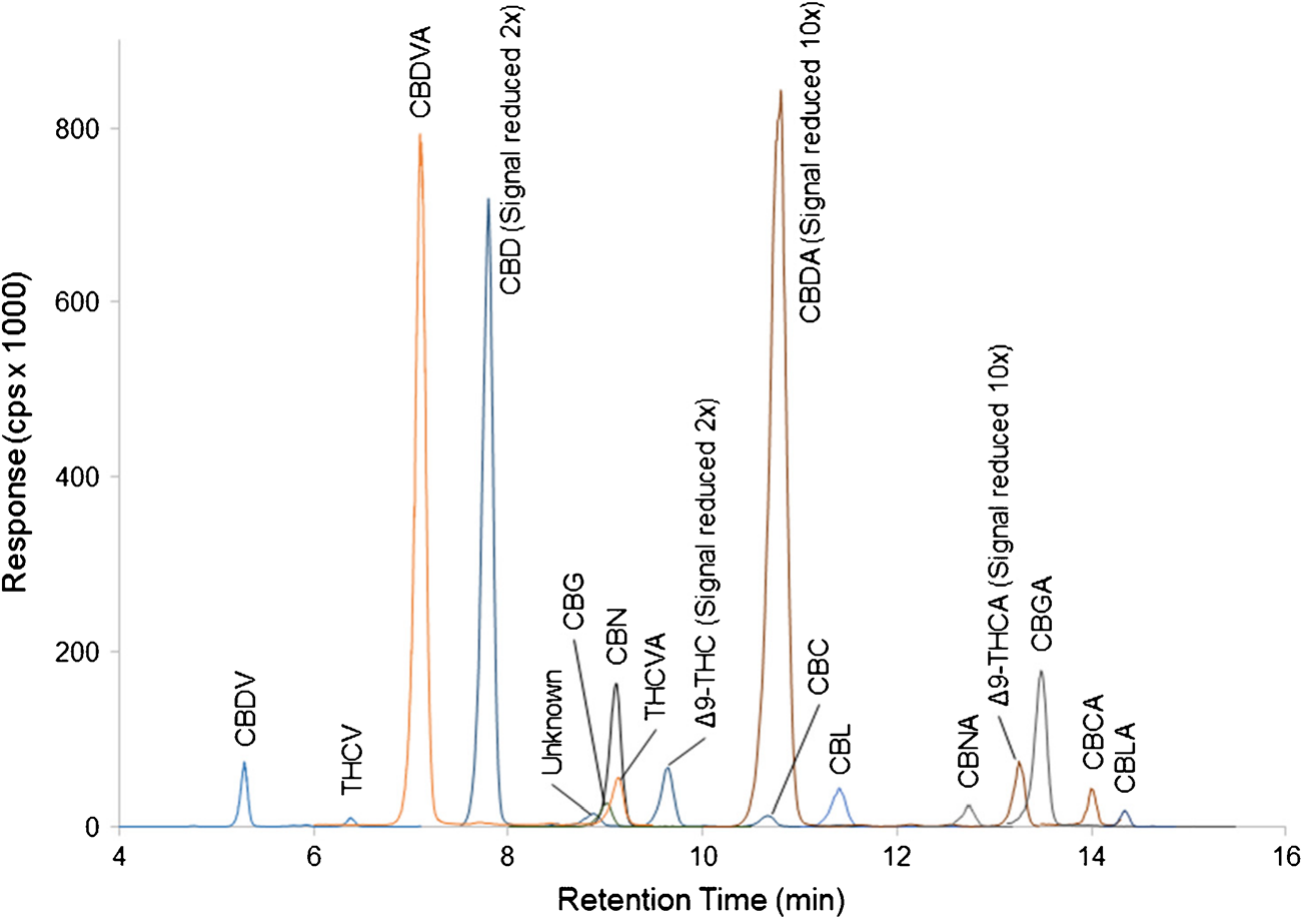
LC-MS/MS chromatogram of 17 cannabinoids in a hemp sample extracted with methanol:water and diluted 1/10 with methanol. The responses of CBD and Δ^9^-THC have been reduced 2-fold while the responses of CBDA and Δ^9^-THCA have been reduced 10-fold to allow visualization of all peaks in the chromatogram

Cannabis and hemp samples were evaluated with the LC-MS/MS conditions. Based on the range of cannabinoid concentrations of the various samples, the cannabis extracts were diluted 1/100, 1/10, and neat, while the hemp samples were diluted 1/10 and neat. Sample chromatograms for representative cannabis and hemp samples are shown in Figs. 3 and 4 respectively.

Due to the high concentration of cannabinoids in matrix and the lack of adequate blank matrices, it is not practical or affordable to spike cannabinoids or internal standards directly into matrix prior to extraction. A common method of quantitation is to prepare calibration curves in solvent and dilute matrix extracts into the calibration curve range. Internal standards may then be added to calibration standards, QC samples, and diluted matrix extracts as the last preparation step. We opted for this approach with calibration curves and cannabis extracts diluted in methanol, and believe it is a reasonable compromise between cost and performance. While only three isotopic derivatives were available commercially at the time of this work, it is recommended that additional internal standards be incorporated into the method as they become available.

### Method validation

#### Specificity/LLOQ

Specificity is difficult to determine without a true cannabis blank matrix; however, the use of LC-MS/MS detection greatly enhances the specificity over LC-UV methods. The inherent specificity of LC-MS/MS is a result of analyte separation and detection by chromatographic retention time and specific molecular ion/product ion selection. The ratio of the quantitation and confirmation ions in the standards and unknown samples were monitored to provide additional specificity. Methanol blanks were evaluated for interfering peaks at the retention time of each cannabinoid and compared to the peak area of the LLOQ (STD-1). The LLOQ for each analyte was required to meet the following criteria: peak areas observed in methanol blanks ≤ 20% of the LLOQ peak area, signal to noise ≥ 10, triplicate preparations of QC-LLOQ provide precision ≤ 20%, and accuracies within ± 20% of nominal concentration. Acceptable carryover was defined as the peak area of a blank methanol sample injected after an upper limit of quantitation standard (ULOQ) providing a peak area ≤ 20% of the LLOQ peak area.

The LLOQ of 10 ng/mL was achieved for all cannabinoids. No peaks or carryover were observed in methanol blanks before or after ULOQ injections and S/N results were ≥ 10 for all cannabinoids. Precision results were ≤ 6.5% and accuracy results were within ± 7.2% of nominal concentration for quadruplicate QC-LLOQ samples for all 17 cannabinoids.

#### Selectivity

Due to its specificity, tandem MS does not require chromatographic separation of all analyte peaks to obtain adequate selectivity. In non-specific detection techniques, analytes should ideally be chromatographically separated with a resolution of 1.5 with 1.0 being the minimum usable separation [28]. In tandem MS detection, analytes with the same mass and product ions (± 2 *m/z*) have the same resolution requirements. For analytes with different masses (≥ 3 *m/z*), or different product ions, any potential isotopic interferences are eliminated. Chromatographic resolution of key cannabinoids was calculated as Resolution = 2(*t*2 − *t*1)/(*W*1 + *W*2) where *t*_1_ and *t*_2_ are the retention times of analyte 1 and 2 respectively and *W*_1_ and *W*_2_ are the baseline peak widths.

Due to the isobaric nature and similarity of structure of many cannabinoids, for example, Δ^9^-THC, Δ^8^-THC, CBD, CBC, and CBL, are all structural isomers and produce the same product ions, chromatographic separation of these and cannabinoids having molecular masses within 2 *m/z* of each other is a requirement of the method. Separation of all key cannabinoid pairs was achieved with peak resolution ranging from 1.0 to 9.5 and is listed in Table S2 (see ESM).

#### Recovery

Recovery was evaluated for a subset of cannabinoids (Δ^9^-THC, Δ^9^-THCA, CBD, CBDA, CBG, CBGA, CBN, CBC, THCV, CBDV, and CBL) via five serial extractions of a cannabis sample of moderate to high Δ^9^-THC and Δ^9^-THCA levels with methanol:water 80:20. The recovery for each extraction was calculated by dividing the area of each extraction by the total area of five serial extractions (adjusted for dilution).

**Fig. 5:**
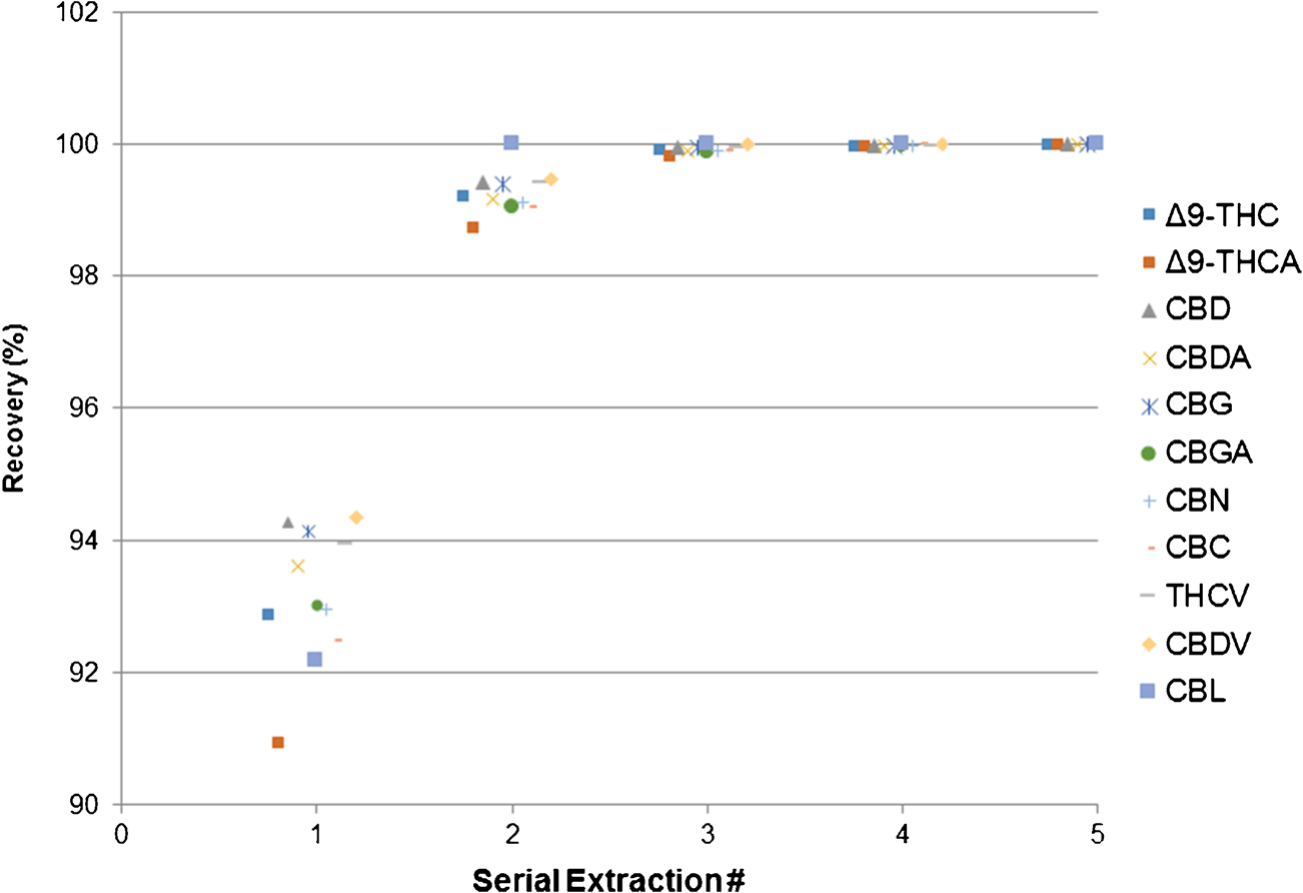
Cumulative percent recovery of a subset of cannabinoids after 1 to 5 serial extractions. Two serial extractions provide recovery ≥ 98.75% while four serial extractions provide recovery ≥ 99.97%

Serial extraction provided excellent recovery for the subset of cannabinoids evaluated. The first extraction resulted in recoveries of 90.9 to 94.3%, while a second extraction recovered an additional 5.5 to 7.8%. A third extraction resulted in an additional 0.0 to 1.1%, a fourth extraction recovered an additional 0.00 to 0.14%, while a fifth extraction recovered an additional 0.00 to 0.025% (Fig. 5). We used four serial extractions to obtain virtually complete recovery, ≥ 99.97%; however, two serial extractions would yield sufficient recovery, ≥ 98.75%, for routine high-throughput methods.

Extraction using methanol:chloroform 90:10, a traditional cannabinoid extraction solvent [12, 22], was also performed to compare to results obtained with methanol:water 80:20. Concentration results for a larger subset of cannabinoids, Δ^9^-THC, Δ^9^-THCA, CBD, CBDA, CBG, CBGA, CBN, CBNA, CBC, CBCA, THCV, THCVA, CBD, and CBDVA, were evaluated. This larger subset of cannabinoids was based on the cannabinoids present in the candidate cannabis NRC certified reference material.

The comparison of extraction using methanol:water 80:20 and methanol:chloroform 90:10 yielded comparable results (within ± 4.3%) for all cannabinoids indicating equivalent performance of the two extraction solvents.

#### Ion suppression

Ion suppression of 14 cannabinoids present in the candidate NRC reference material and 3 internal standards was evaluated by spiking cannabinoids into diluted matrix extracts to yield double the incurred concentration of the extract. The pure cannabinoid solutions at 1× incurred matrix concentration (Sample1) and 2× incurred concentration (Sample4) as well as the un-spiked diluted extract (Sample2) and spiked diluted extract (Sample3) were then analyzed. Sample1 and Sample4 were used to determine cannabinoid response factor (RF) in pure solution: $\mathrm{RF}=2\times \Big(\frac{\mathrm{peak}\ \mathrm{area}\ \mathrm{Sample}1}{\mathrm{peak}\ \mathrm{area}\ \mathrm{Sample}4}$).

% Ion suppression was then calculated as follows:

$$ \%\mathrm{Ion}\ \mathrm{Suppression}=1-\Big(\frac{\left(\mathrm{peak}\ \mathrm{area}\ \mathrm{Sample}3\ast \mathrm{RF}\right)-\mathrm{peak}\ \mathrm{area}\ \mathrm{Sample}2}{\mathrm{peak}\ \mathrm{area}\ \mathrm{Sample}1} $$).

Results were expressed in %, with positive values indicating ion suppression and negative values indicating ion enhancement. Ion suppression was deemed to be acceptable if % suppression or enhancement were within ± 10%.

The results ranged from − 5.5 to 4.4% indicating no significant ion suppression or enhancement was observed. The results validate the approach to use external calibration curves spiked in methanol for the quantitation of extracts.

#### Linearity

The calibration curves consisted of ten non-zero standards prepared in methanol. Linear regressions, weighted 1/concentration^2^ to extend the linear range [32] and reduce the number of dilutions required, were generated using the peak area ratios (analyte/internal standard) versus the calibration standard concentrations. As isotopic derivatives were not available for all cannabinoids, Table 1 lists which internal standards were used to quantify each cannabinoid. Note that CBN-d3 was used in most cases other than Δ^9^-THC/THCA and CBD/CBDA due to its moderate retention time and similar levels to several minor cannabinoids. Correlation coefficients (*r*^2^) were calculated using Xcalibur software V4.0.27.10 for each cannabinoid. Linearity was evaluated over three batches on 3 separate days and acceptance criteria were set as follows: correlation coefficient ≥ 0.99 and visual examination to indicate linearity. All calibration curves met this criteria, as shown in Figs. S1 to S17 (see ESM). The large calibration range (1000-fold) allowed for a greater number of sample extracts to fall within the calibration range.

#### Quality control sample precision and accuracy

Quadruplicate preparations of the QC samples (QC-LLOQ, QC-1, QC-2, and QC-3) were evaluated in batches prepared on three separate days. Within-batch precision and accuracy were determined for the first batch and between-batch precision and accuracy were determined for the three batches combined. Acceptance criteria were set as follows: precision ≤ 15% (20% for QC-LLOQ) and accuracy within ± 15% (20% for QC-LLOQ) of nominal concentration.

Within-batch precision of the quality control samples (QC-LLOQ, QC-1, QC-2, and QC-3) for all 17 cannabinoids ranged from 0.5 to 6.5% while within-batch accuracy ranged from 91.4 to 108.0%.

Between-batch precision of the quality control samples ranged from 0.9 to 5.1% while between-batch accuracy ranged from 91.5 to 107.5%.

#### Precision and accuracy using NRC reference material

Single preparations of the NRC candidate reference material were evaluated via triplicate injections in batches prepared on three separate days. Within-batch precision and accuracy were determined for the first batch and between-batch precision and accuracy were determined for the three batches combined. Acceptance criteria were set as follows: precision ≤ 15% and accuracy within ± 15% of nominal concentration.

Within-batch precision for 14 cannabinoids (Δ^9^-THC, Δ^9^-THCA, CBD, CBDA, CBG, CBGA, CBN, CBNA, CBC, CBCA, THCV, THCVA, CBD, and CBDVA) ranged from 0.2 to 3.6% while within-batch accuracy ranged from 85.4 to 111.6%. Between-batch precision ranged from 1.4 to 6.1% while between-batch accuracy ranged from 90.2 to 110.3%.

#### Quality control sample stability

The stability of QC samples prepared in methanol was evaluated for QCs stored at − 20 °C for 6 months. Fresh preparations of QC-1, QC-2, and QC-3 samples as well as the 6-month stability QC samples were analyzed in quadruplicate in a single batch with results reported as % difference. QCs were determined to be stable if the % difference between fresh and stability QCs was ≤ 10%.

All stability and freshly prepared QC samples met batch acceptance criteria with % differences between stability and fresh QC results ranging from − 5.9 to 4.9%. The results indicate that QC samples are stable at − 20 °C for 6 months.

#### Extract stability

Extract stability for undiluted sample extracts in methanol:water 80:20 was evaluated at − 20 °C. Triplicate samples were extracted and analyzed (Time 0) and stored at − 20 °C for 8 weeks. The samples were re-analyzed against a fresh calibration curve and the time 0 results were compared to the 8 week stability results and reported as % difference. Extracts were determined to be stable if the % difference between time 0 and stability results was ≤ 10%.

Triplicate analysis of sample extracts after storage at − 20 °C for 8 weeks produced results within ± 6.7% of the time 0 results for 14 cannabinoids evaluated. The results indicate that the sample extracts may be stored at − 20 °C for up to 8 weeks.

#### Processed sample stability

Processed sample stability was evaluated by reinjecting a batch after storage of the samples at 4 °C for 15 days. The calibration curve and QC samples were verified against acceptance criteria and the initial and stability results for the seven cannabis samples were compared with results reported as average % difference. Batch samples were determined to be stable if the average % difference between time 0 and stability results was ≤ 10%.

Reanalysis of a batch stored at 4 °C for 15 days provided QC sample results within acceptance criteria with precision and accuracy ranging from 0.5 to 6.3% and 90.3 to 107.8% respectively for the 17 cannabinoids. The average % difference for sample results between the first result and the stability result ranged from − 7.6 to 5.0%. The results indicate that the processed samples may be stored at 4 °C for 15 days.

#### Application to cannabis and hemp samples

Seven cannabis samples were selected to evaluate the method with samples representing a range of THC and CBD concentrations. Two samples with label claims for medium, balanced THC/CBD concentrations, two with high THC/low CBD concentrations, two with low THC/high CBD concentrations, and one with unknown concentrations, were extracted and analyzed in triplicate. While three dilution levels (1/100, 1/10, and neat) were analyzed for each cannabis sample, only the average of the 1/100 and/or 1/10 dilutions (triplicate samples) was reported if a valid result was obtained. A valid result was defined as a result within the calibration curve range. The neat extract sample results were reported only if a valid result was not obtained for the 1/100 and/or 1/10 diluted samples. Including all three dilution levels (1/100, 1/10, and neat), the quantifiable range was from 0.002 to 200 mg/g in matrix, representing a 100,000-fold range.

Sample results (average of triplicate analysis on three separate days) were compared to label claims for THC, total THC, CBD, and total CBD. Label claims did not specify THCA or CBDA as individual results. Total cannabinoid concentrations in mg/g were determined as neutral equivalent as follows:

$$ \mathrm{Total}=\mathrm{Neutral}\ \mathrm{conc}+\mathrm{Acid}\ \mathrm{conc}\times \left(\frac{\mathrm{MW}\ \mathrm{neutral}}{\mathrm{MW}\ \mathrm{acid}}\right) $$

Hemp samples, due to their lower overall expected cannabinoid results, were diluted 1/10 with analysis of the neat and 1/10 diluted samples. Five lots of the same larger hemp sample were evaluated with triplicate analysis of each lot. All sample results were reported in mg/g for alignment with the International System of Units (SI) and to avoid confusion when discussing percent cannabinoid levels in combination with percent relative standard deviation (RSD).

While the 1000-fold calibration range allowed many sample results (Table 2) to fall within range, a single dilution was not sufficient for all cannabinoids in all cannabis samples due to the very large concentration range observed (< 0.002 to 166 mg/g). Seven cannabis samples were analyzed for 17 cannabinoids for a total of 119 potential results. Δ^8^-THC was not observed in any of the cannabis samples and CBL and CBLA, when observed, were at low concentrations (≤ 0.022 mg/g). A 1/100 dilution of the extracts allowed quantitation of samples from 0.2 to 200 mg/g (63 valid results) while a 1/10 dilution of the extracts allowed quantitation of samples from 0.02 to 20 mg/g (80 valid results). Analysis of the neat extracts allowed quantitation of samples from 0.002 to 2 mg/g (11 results reported), while 21 results were reported as below the limit of quantitation (BLQ) of 0.002 mg/g for all dilutions. No results were observed above the limit of quantitation (ALQ) for the 1/100 diluted samples.

**Table 2:** Repeatability and intermediate precision of cannabinoids in cannabis

	ID	^Δ9−^THC	^Δ9−^THCA	CBD	CBDA	CBG	CBGA	CBN	CBNA	CBC	CBCA	THCV	THCVA	CBDV	CBDVA	CBL	CBLA
Conc (mg/g)	1	6.26	51.4	4.37	93.2	0.721	3.15	0.123	0.845	0.422	5.25	0.051	0.407	0.020	0.428	BLQ	0.00914
Repeat (%)		9.2	7.6	8.9	8.8	7.1	9.8	6.5	5.5	3.9	9.8	6.2	7.8	1.8	6.7	N/AP	7.8
Int Prec (%)		7.1	6.1	8.5	5.9	7.1	7.8	5.6	4.3	5.0	6.9	5.3	6.5	6.8	6.5	N/AP	8.0
HorRat		1.7	2.0	1.9	2.1	1.2	1.6	0.7	0.7	0.8	1.6	0.6	1.0	0.7	1.0	N/AP	0.7
Conc (mg/g)	2	26.9	26.0	18.1	67.6	0.474	1.80	0.02	0.762	1.46	4.08	0.204	0.210	0.0700	0.282	0.0021	0.0218
Repeat (%)		3.6	4.6	3.6	4.0	3.5	4.3	5.1	3.6	4.9	4.5	4.9	3.3	6.2	4.1	8.5	10.0
Int Prec (%)		4.4	3.9	4.0	3.7	4.2	4.1	4.2	5.2	5.7	5.9	4.6	3.7	4.9	4.5	10.0	9.3
HorRat		1.3	1.1	1.1	1.2	0.7	0.8	0.7	0.9	1.1	1.3	0.6	0.5	0.6	0.7	0.7	0.9
Conc (mg/g)	3	6.92	124	0.010	0.275	0.726	2.18	0.155	2.36	0.092	0.973	0.034	0.569	BLQ	0.0024	BLQ	BLQ
Repeat (%)		3.4	4.7	4.4	5.4	4.0	5.3	4.5	4.6	2.0	10.3	3.1	2.6	N/AP	5.4	N/AP	N/AP
In. Prec (%)		6.1	5.9	8.1	7.6	7.4	8.0	11.0	7.4	8.8	10.7	8.0	6.8	N/AP	11.9	N/AP	N/AP
HorRat		1.4	2.1	0.7	1.1	1.2	1.6	1.5	1.5	1.1	1.9	0.9	1.1	N/AP	0.9	N/AP	N/AP
Conc (mg/g)	4	5.27	166	0.00736	0.361	1.05	3.83	0.0207	0.515	0.159	3.04	0.0133	0.279	BLQ	BLQ	BLQ	BLQ
Repeat (%)		3.1	2.1	8.8	2.8	1.7	2.2	1.5	2.6	2.9	5.1	12.4	3.8	N/AP	N/AP	N/AP	N/AP
Int Prec (%)		8.9	8.1	12.8	6.5	7.2	8.1	7.4	8.7	5.5	7.0	11.0	7.1	N/AP	N/AP	N/AP	N/AP
HorRat		2.0	3.1	1.1	1.0	1.3	1.7	0.7	1.4	0.7	1.5	1.0	1.0	N/AP	N/AP	N/AP	N/AP
Conc (mg/g)	5	0.917	4.89	9.26	141	0.391	1.29	0.0240	0.0643	0.678	6.49	0.0103	0.0576	0.0830	1.38	BLQ	BLQ
Repeat (%)		6.5	4.0	5.6	6.3	4.7	7.5	1.2	7.0	7.7	7.0	5.8	2.2	1.9	5.9	N/AP	N/AP
Int Prec (%)		5.2	3.8	5.5	4.4	3.8	5.4	3.6	5.1	5.4	5.5	4.4	2.2	3.6	4.8	N/AP	N/AP
HorRat		0.9	0.9	1.4	1.6	0.6	1.0	0.4	0.6	0.9	1.3	0.4	0.3	0.4	0.9	N/AP	N/AP
Conc (mg/g)	6	0.803	5.16	9.31	151	0.636	2.53	0.0356	0.113	0.755	7.91	0.00267	0.0157	0.0226	0.362	0.0079	0.0655
Repeat (%)		2.6	3.2	3.4	1.6	3.6	3.1	3.9	4.5	2.7	5.4	5.6	3.3	5.2	4.4	3.6	3.7
Int Prec (%)		4.6	4.5	5.7	3.8	4.3	4.8	4.2	4.2	4.9	5.2	8.2	8.8	5.6	4.3	3.7	10.8
HorRat		0.8	1.0	1.4	1.4	0.7	1.0	0.4	0.4	0.8	1.2	0.6	0.8	0.6	0.7	0.3	1.3
Conc (mg/g)	7	6.69	13.9	0.00867	0.0462	0.0634	0.380	0.129	0.251	0.284	0.551	0.0436	0.104	BLQ	BLQ	BLQ	BLQ
Repeat (%)		4.8	4.8	6.8	8.1	5.3	4.6	7.6	7.2	3.9	7.8	6.0	6.1	N/AP	N/AP	N/AP	N/AP
Int Prec (%)		5.5	4.9	8.7	6.3	4.3	5.4	4.6	6.3	4.4	5.8	4.8	4.7	N/AP	N/AP	N/AP	N/AP
HorRat		0.3	0.3	0.8	0.7	0.5	0.8	0.6	0.9	0.6	0.9	0.5	0.6	N/AP	N/AP	N/AP	N/AP

The results obtained for Δ^9^-THC, total Δ^9^-THC, CBD, and total CBD were compared to the label claims of the six cannabis samples that had concentrations listed (Table 3). Not surprisingly, the Δ^9^-THC and CBD levels determined were significantly higher (33.8 to 229%) than the label claims (with the exception of CBD for sample 3). The higher observed neutral levels can be attributed to storage of the products at room temperature and the well-known conversion of cannabinoid acid form to neutral form over time [33, 34]. The discrepancy in the CBD result for sample 3 is suspected to be due to an overestimate of the CBD concentration by the testing laboratory, likely due to a misidentified peak or an interfering peak in the UV chromatogram. Eight of the 12 results for total Δ^9^-THC and total CBD results were within ± 15% of the label claim while two total CBD results were not able to be evaluated due to the reported concentrations of 0.0 mg/g. These results were found to be 0.252 and 0.324 mg/g using our method, which is likely BLQ for the supplier’s method. Two results for total Δ^9^-THC were 24–29% lower than label claim. The reason for these discrepancies is not known; however, possibilities include errors in the calibration or interfering peaks.

**Table 3:** THC and CBD results compared to label claims for six cannabis products obtained from the regulated market in Canada through the Ontario Cannabis Store

ID	THC label (mg/g)	THC LC-MS (mg/g)	THC difference %	Total THC label (mg/g)	Total THC LC-MS (mg/g)	Total THC difference %
1	1.9	6.26	229	58.0	51.4	− 11.4
2	15.4	26.9	74.4	70.0	49.7	− 29.0
3	3.7	6.92	87.1	152	115	− 24.1
4	1.7	5.27	210	156	151	− 3.4
5	0.5	0.917	83.5	6.0	5.21	− 13.2
6	0.6	0.803	33.8	6.0	5.33	− 11.2
ID	CBD label (mg/g)	CBD LC-MS (mg/g)	CBD difference %	Total CBD label (mg/g)	Total CBD LC-MS (mg/g)	Total CBD difference %
1	1.6	4.37	173	96.0	86.2	− 10.2
2	8.9	18.1	104	90.0	77.4	− 14.0
3	0.5	0.0100	− 98.0	0.0	0.252	N/AP
4	0.0	0.00726	N/AP	0.0	0.324	N/AP
5	4.3	9.26	115	133	133	− 0.1
6	5.7	9.31	63.3	147	142	− 3.6

Inspection of the results for THCV/THCVA and CBDV/CBDVA revealed that, while relatively much lower, concentrations appear to correlate with the concentrations of Δ^9^-THC/Δ^9^-THCA and CBD/CBDA respectively. Structurally, the difference is a 3-carbon side-chain for THCV/CBDV versus a 5-carbon side-chain for Δ^9^-THC/CBD so this is not an unexpected observation. The results for CBN did not appear to correlate well with THC concentrations; however, a weak correlation was observed for CBNA relative to Δ^9^-THCA.

Results for all cannabinoids in hemp samples were determined with the 1/10 and neat sample dilutions with the average of valid triplicate results reported in Table 4. Review of the data indicated that total THC concentrations were below the 3 mg/g (0.3%) level to be considered legal hemp in some jurisdictions and that CBD, CBDA, and all of the minor cannabinoids included in the method were observed and quantified.

**Table 4:** Hemp sample cannabinoid results and repeatability

	ID	^Δ9−^THC	^Δ9−^THCA	CBD	CBDA	CBG	CBGA	CBN	CBNA	CBC	CBCA	THCV	THCVA	CBDV	CBDVA	CBL	CBLA
Conc (mg/g)	1	0.365	0.986	4.84	13.81	0.049	0.119	0.377	0.268	0.252	0.459	0.017	0.074	0.159	0.749	0.057	0.181
Repeat (%)		5.0	1.9	0.3	0.8	13.7	8.7	4.9	4.4	1.7	6.7	1.7	8.6	8.3	6.4	9.3	8.4
Conc (mg/g)	2	0.377	0.968	5.235	14.18	0.051	0.119	0.399	0.281	0.267	0.473	0.017	0.074	0.175	0.759	0.062	0.184
Repeat (%)		3.3	4.1	0.5	0.7	11.3	7.0	3.3	5.9	2.9	3.5	1.4	5.9	7.0	5.3	8.3	7.8
Conc (mg/g)	3	0.400	1.08	5.89	15.80	0.054	0.128	0.402	0.299	0.287	0.521	0.018	0.079	0.192	0.813	0.064	0.205
Repeat (%)		2.5	7.5	3.0	1.9	7.3	2.8	1.4	13.1	8.0	6.9	0.5	2.5	3.7	1.6	4.5	8.2
Conc (mg/g)	4	0.437	1.149	6.42	16.73	0.059	0.140	0.456	0.333	0.314	0.562	0.020	0.087	0.220	0.899	0.072	0.219
Repeat (%)		2.0	4.2	1.3	1.3	9.1	6.0	3.3	8.8	5.9	4.7	1.0	4.8	4.7	3.4	8.0	9.8
Conc (mg/g)	5	0.481	1.262	6.56	17.48	0.063	0.154	0.457	0.357	0.325	0.585	0.022	0.098	0.229	0.956	0.074	0.232
Repeat (%)		4.0	1.7	0.4	0.8	10.7	6.8	4.8	4.9	2.8	5.8	0.8	5.5	6.5	6.0	8.7	8.2
Av Conc (mg/g)		0.412	1.089	5.81	15.60	0.055	0.131	0.418	0.308	0.289	0.520	0.017	0.082	0.195	0.835	0.065	0.202
Overall Repeat (%)		10.9	10.9	11.6	9.5	13.5	12.1	8.6	13.2	10.7	10.9	11.5	12.5	14.7	10.8	12.6	12.7

#### Sample repeatability, intermediate precision, and HorRat

Triplicate extractions of cannabis samples were analyzed on a single day to evaluate method repeatability (% RSD). This process was repeated on three separate days to evaluate intermediate precision (% RSD) while the overall method precision was evaluated via calculation of the HorRat values [35].

Repeatability was evaluated using results from triplicate analysis of seven different cannabis samples on Day-1 and is presented in Table 2. Repeatability RSDs ranged from 1.2 to 12.4%. The higher RSD values for Sample-1 were found to be due to differences between triplicate extractions of samples. This effect is most likely due to inhomogeneity of the ground sample as the effect was observed for all cannabinoids within the affected sample replicates.

Intermediate precision was evaluated using the combined triplicate results for cannabinoids analyzed on three different days and is presented in Table 2. Intermediate precision RSDs ranged from 2.2 to 12.8%.

The Horwitz ratio (HorRat) is a measure of the acceptability of % RSD based on sample concentration and was calculated for all cannabinoids and valid sample results (Table 2). The HorRats ranged from 0.3 to 3.1 with 3 of 117 valid results falling above 2.0 and 8 of 117 results falling below 0.5. The 3 values above 2.0 were the 3 highest concentration results observed, indicating that the high HorRats were more a factor of the higher concentrations than higher RSDs. The 8 values below 0.5 were low concentration values combined with low RSDs.

Only repeatability was determined for the hemp samples as the analysis was only performed once. The repeatability for individual lots of hemp ranged from 0.1 to 13.7% with only 4 results (three CBG and one CBNA), ≥ 10.0%. The overall repeatability (all results combined for all lots) ranged from 8.6 to 14.7%. Review of the data indicated that the higher repeatability values for the combined data were a result of slight concentration differences between the hemp lots.

## Conclusions

The rapid increase in demand for the analysis of cannabinoids in cannabis and hemp has resulted in a similar demand for analytical methods that are able to meet the current regulatory requirements and be adaptable to future requirements such as cannabinoid analysis in edibles. HPLC-UV has been and continues to be a heavily used technique to meet this demand; however, it has limitations with respect to sensitivity, specificity, and peak separation requirements. LC-MS/MS has become a common analytical technique in many laboratories due to its superior sensitivity, specificity, and less stringent peak separation requirements. Additional cannabinoids, as standards become available, can be added more easily to LC-MS/MS methods than to HPLC-UV methods which require complete chromatographic separation of all cannabinoids. The low limits of quantitation and wide range of quantitation in matrix reported here for 17 cannabinoids are made possible by the advantages provided by LC-MS/MS. We expect that this method will be easily adapted to more challenging matrices such as oils and edibles [18, 36] providing the opportunity to utilize a consistent technique for all matrices, making it a logical choice for cannabinoid analysis.

In summary, we have developed and validated a LC-MS/MS method capable of quantifying 17 cannabinoids in cannabis and hemp over a range of 0.002 to 200 mg/g (0.0002 to 20%). The method has demonstrated sensitivity, specificity, accuracy, precision, and stability during analysis of cannabis and hemp samples and can be adapted to concentration ranges and additional matrices as required.

While LC-MS/MS is not yet widely used for routine analysis of cannabinoids in cannabis and hemp, we believe that the application of LC-MS/MS methods will lead to significant improvements in data quality and consistency between laboratories. This enhancement in measurement comparability will increase confidence of both consumers and regulators and ultimately benefit regulated cannabis markets.

## Electronic supplementary material

ESM 1 (PDF 206 kb)
