# Supplementary material for: Quantitative determination and validation of 17 cannabinoids in cannabis and hemp using liquid chromatography-tandem mass spectrometry
Source: Anal Bioanal Chem. 2020 Aug 24;412(27):7381–93. doi: 10.1007/s00216-020-02862-8 (PMC7533253; doi:10.1007/s00216-020-02862-8)
Supplement: Supplementary file 1 — (PDF 206 kb) [file 216_2020_2862_MOESM1_ESM.pdf]

Analytical and Bioanalytical Chemistry

Electronic Supplementary Material

Quantitative determination and validation of 17 cannabinoids in cannabis and hemp using liquid chromatography−mass spectrometry

Garnet McRae, Jeremy E. Melanson

Table of Contents

List of Tables

Table S1 Calibration standard and QC sample concentrations

Table S2 Chromatographic peak resolution

List of Figures

Figure S1 Δ9-THC linear regression

Figure S2 Δ9-THCA linear regression

Figure S3 CBD linear regression

Figure S4 CBDA linear regression

Figure S5 CBG linear regression

Figure S6 CBGA linear regression

Figure S7 CBN linear regression

Figure S8 CBNA linear regression

Figure S9 CBC linear regression

Figure S10 CBCA linear regression

Figure S11 THCV linear regression

Figure S12 THCVA linear regression

Figure S13 CBDV linear regression

Figure S14 CBDVA linear regression

Figure S15 CBL linear regression

Figure S16 CBLA linear regression

Figure S17 Δ8-THC linear regression

Table S1 Calibration standard and QC sample concentrations (prepared in methanol, concentrations for each of 17 cannabinoids for each standard level)

Standard / QC Sample ID	Cannabinoid Concentration (ng/mL)
STD-10	10 000
STD-9	9 000
STD-8	6 000
STD-7	2 000
STD-6	1 000
STD-5	400
STD-4	100
STD-3	40
STD-2	20
STD-1	10
STD-0	0
QC-3	8 000
QC-2	1 500
QC-1	30
QC-LLOQ	10

Table S2 Chromatographic peak resolution of key cannabinoids within 2 *m/z* of each other

Cannabinoid-1	Cannabinoid-2	Δ *m/z*	Resolution
CBDV	THCV	0	5.8
CBD	Δ9-THC	0	7.2
Δ9-THC	Δ8-THC	0	1.9
Δ8-THC	CBC	0	1.9
CBC	CBL	0	2.4
CBD	CBG	2	4.7
CBG	Δ9-THC	2	2.1
CBDVA	THCVA	0	7.4
CBDA	Δ9-THCA	0	8.5
Δ9-THCA	CBCA	0	3.6
CBCA	CBLA	0	2.1
CBDA	CBGA	2	9.5
Δ9-THCA	CBGA	2	1.0

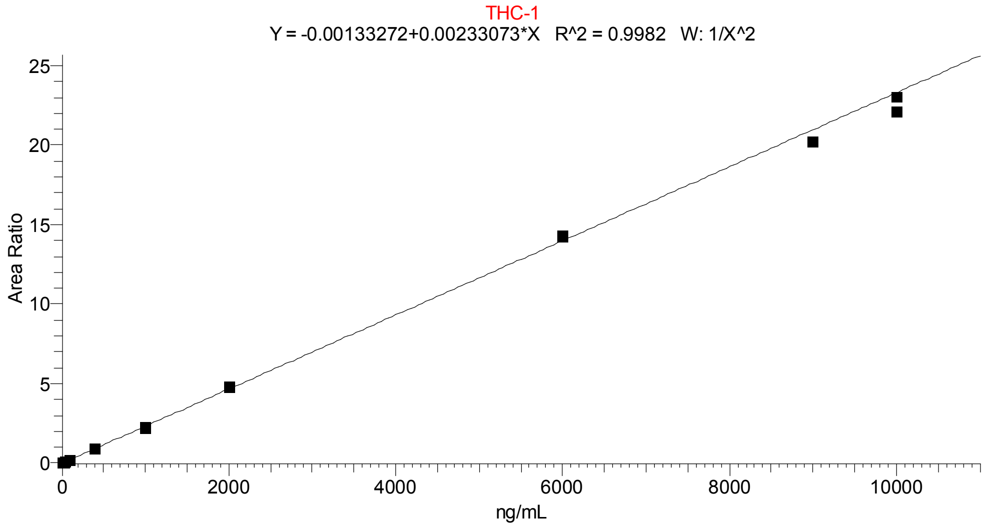

Fig. S1 Δ9-THC Linear regression, weighted $1/x^2$ (duplicate injection of a calibration curve, beginning and end of batch)

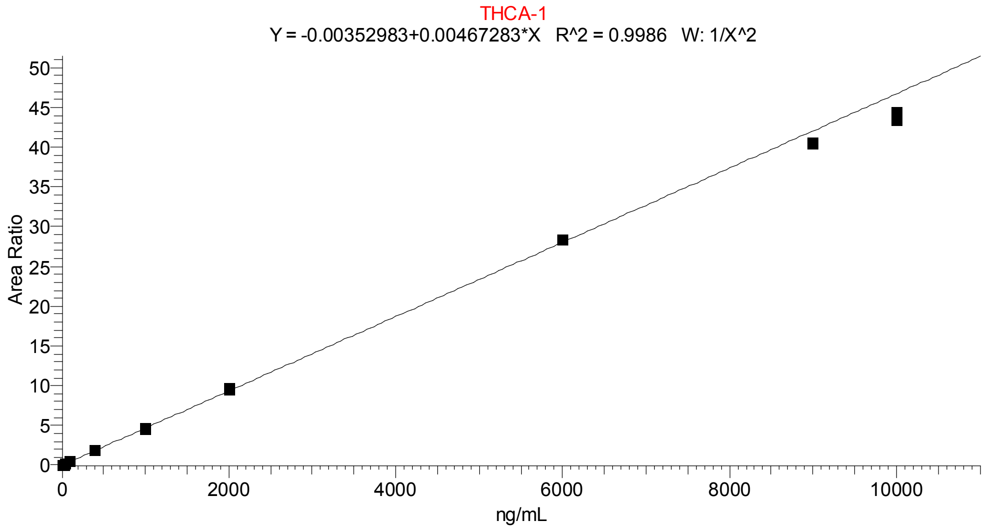

Fig. S2 Δ9-THCA Linear regression, weighted $1/x^2$ (duplicate injection of a calibration curve, beginning and end of batch)

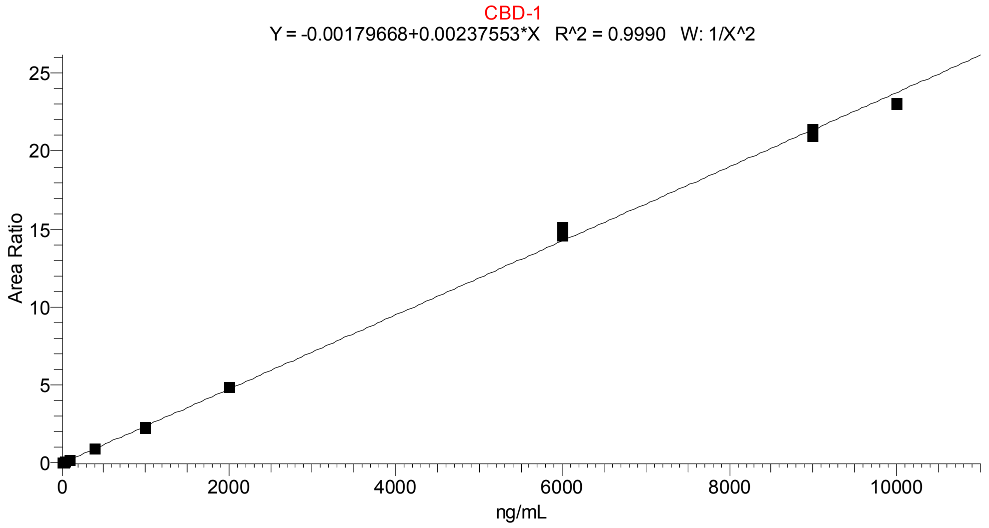

Fig. S3 CBD Linear regression, weighted $1/x^2$ (duplicate injection of a calibration curve, beginning and end of batch)

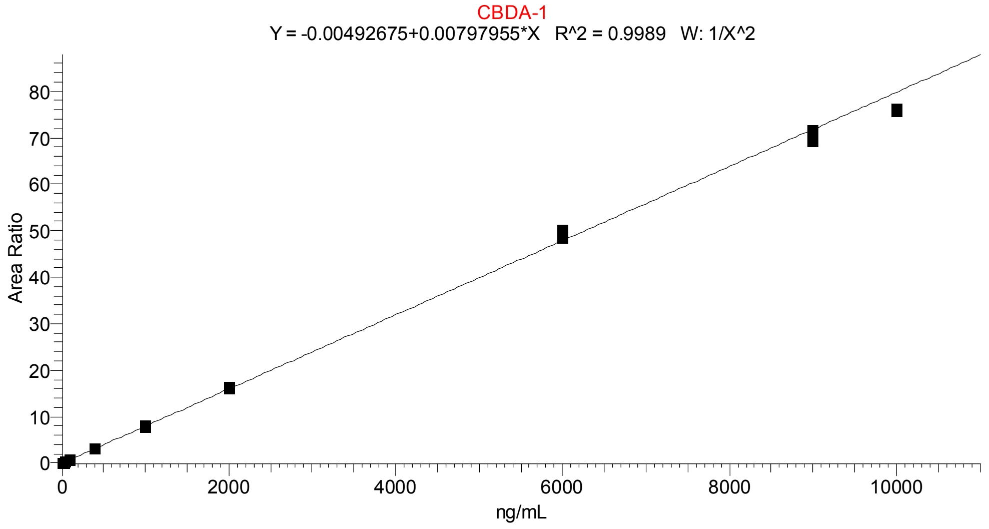

Fig. S4 CBDA Linear regression, weighted $1/x^2$ (duplicate injection of a calibration curve, beginning and end of batch)

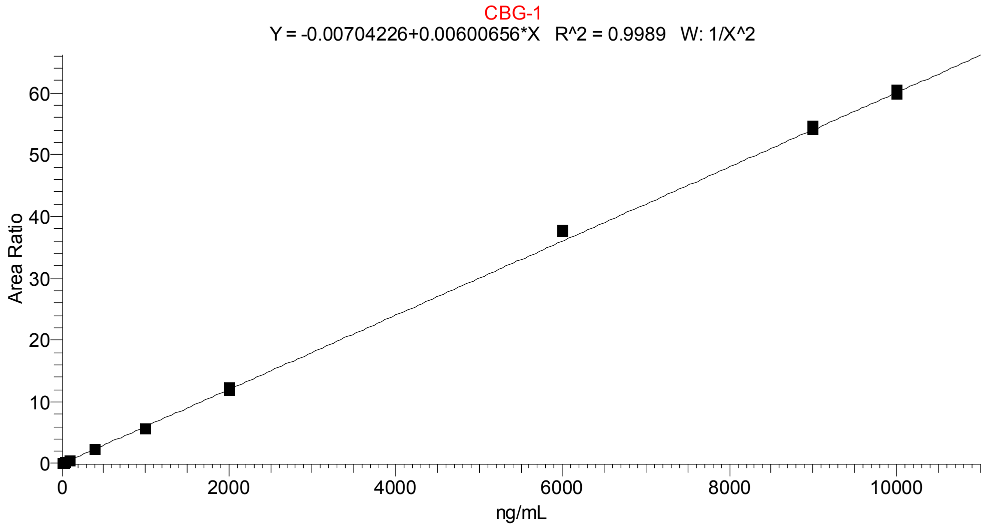

Fig. S5 CBG Linear regression, weighted $1/x^2$ (duplicate injection of a calibration curve, beginning and end of batch)

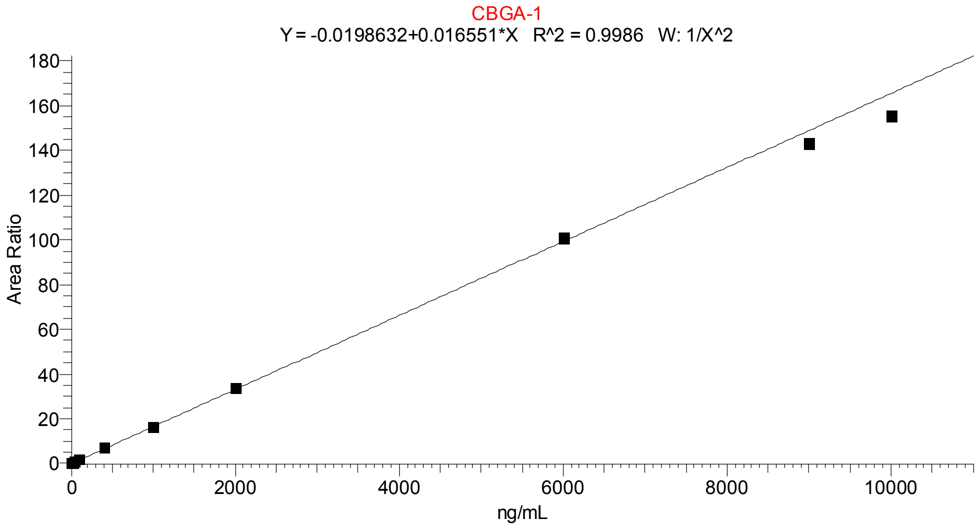

Fig. S6 CBGA Linear regression, weighted $1/x^2$ (duplicate injection of a calibration curve, beginning and end of batch)

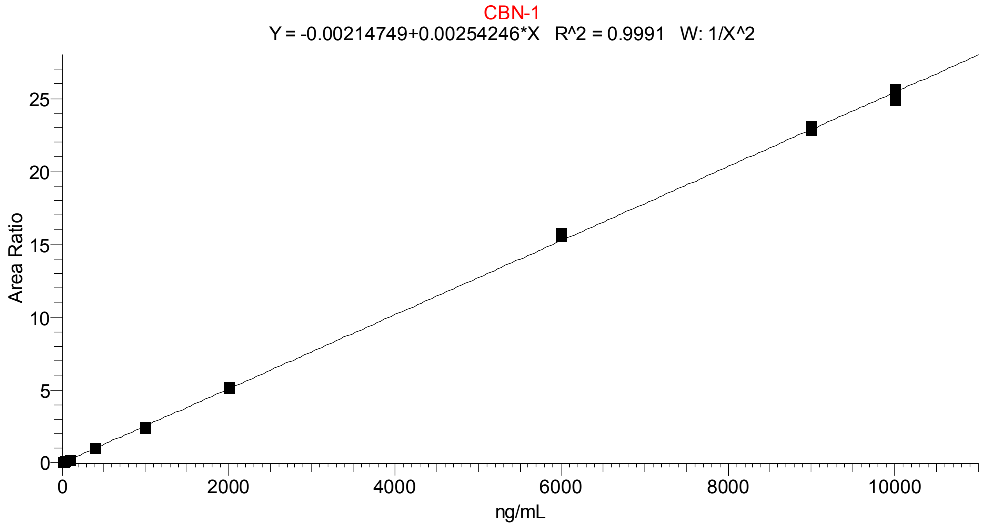

Fig. S7 CBN Linear regression, weighted $1/x^2$ (duplicate injection of a calibration curve, beginning and end of batch)

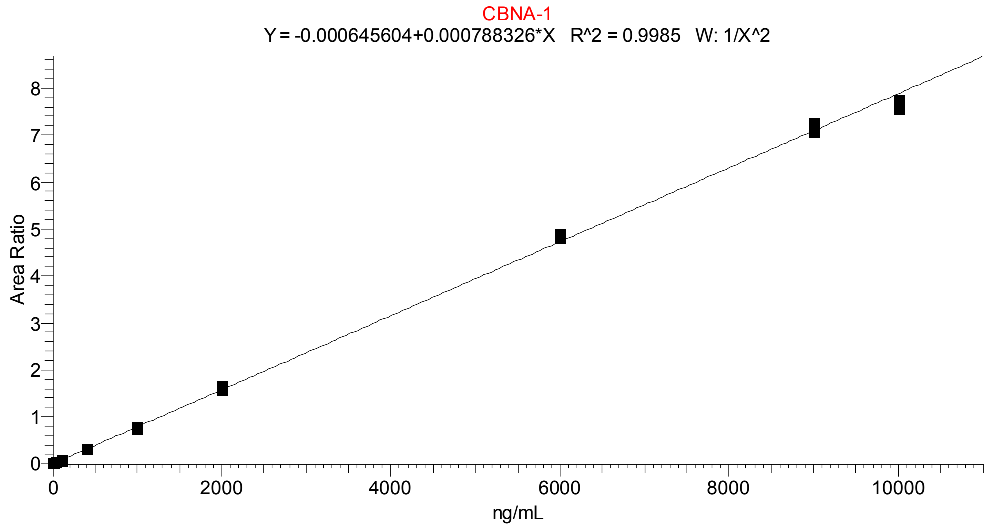

Fig. S8 CBNA Linear regression, weighted $1/x^2$ (duplicate injection of a calibration curve, beginning and end of batch)

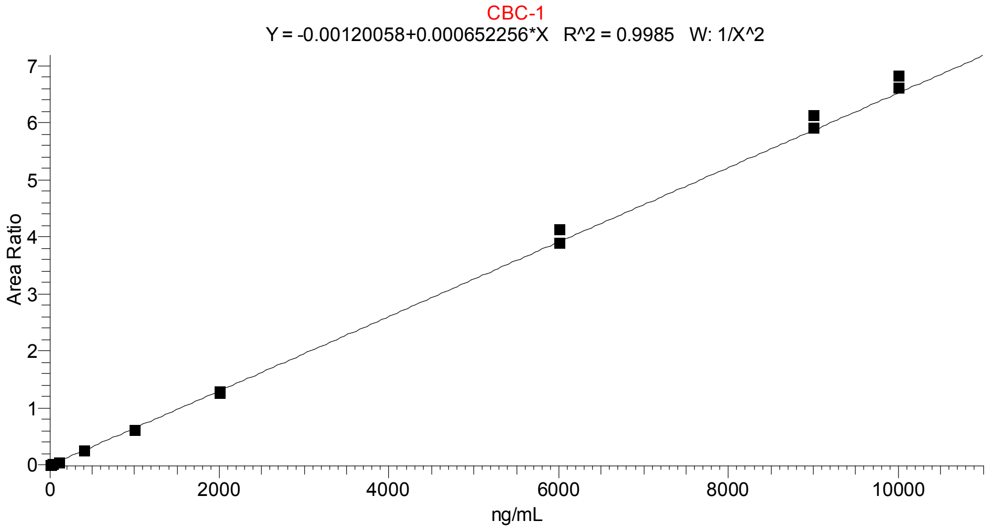

Fig. S9 CBC Linear regression, weighted $1/x^2$ (duplicate injection of a calibration curve, beginning and end of batch)

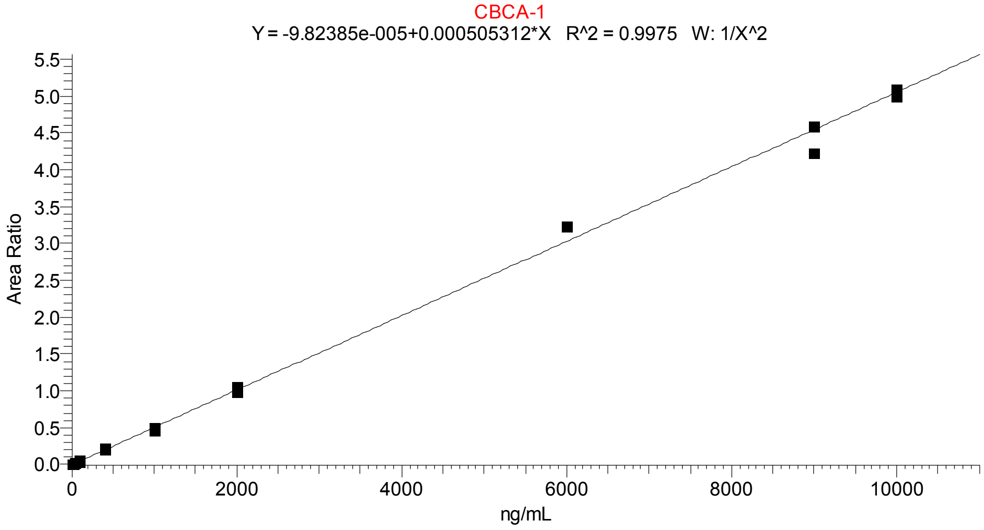

Fig. S10 CBCA Linear regression, weighted $1/x^2$ (duplicate injection of a calibration curve, beginning and end of batch)

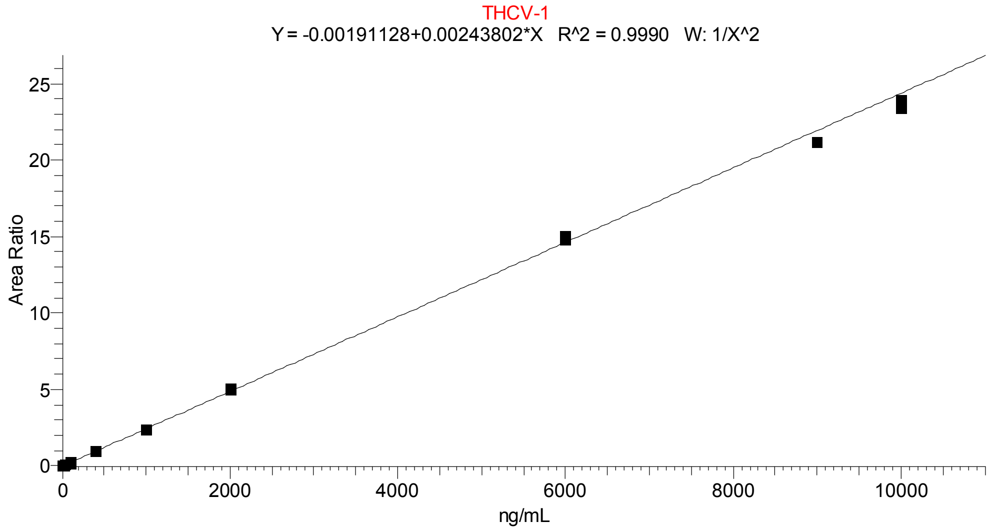

Fig. S11 THCV Linear regression, weighted $1/x^2$ (duplicate injection of a calibration curve, beginning and end of batch)

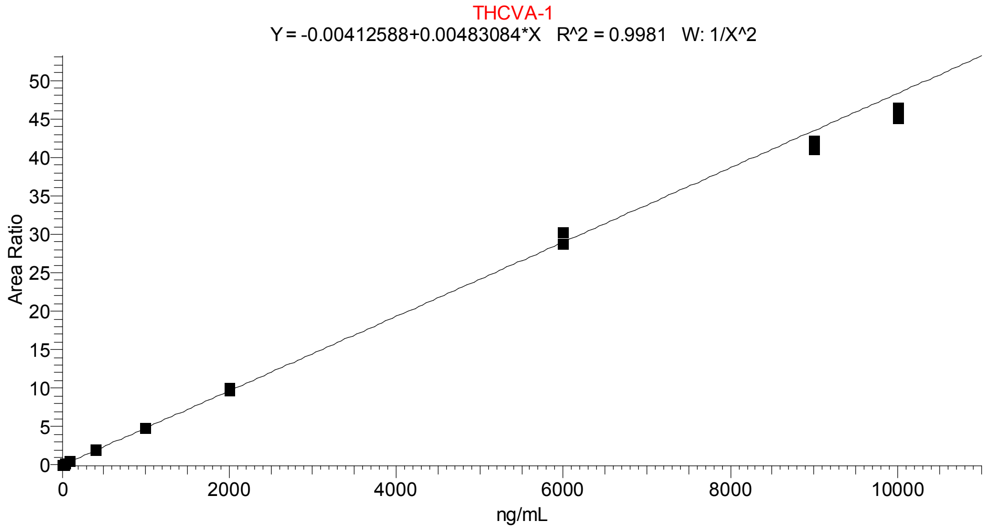

Fig. S12 THCVA Linear regression, weighted $1/x^2$ (duplicate injection of a calibration curve, beginning and end of batch)

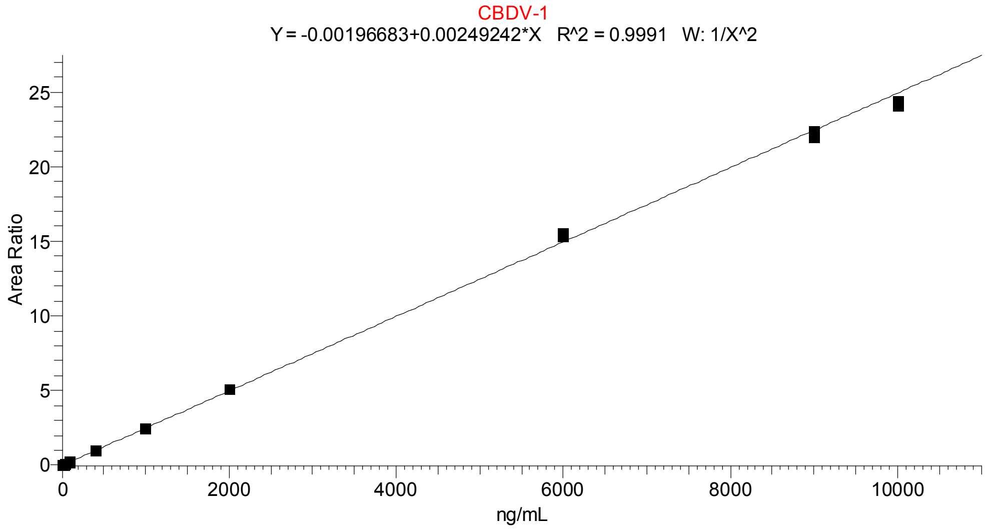

Fig. S13 CBDV Linear regression, weighted $1/x^2$ (duplicate injection of a calibration curve, beginning and end of batch)

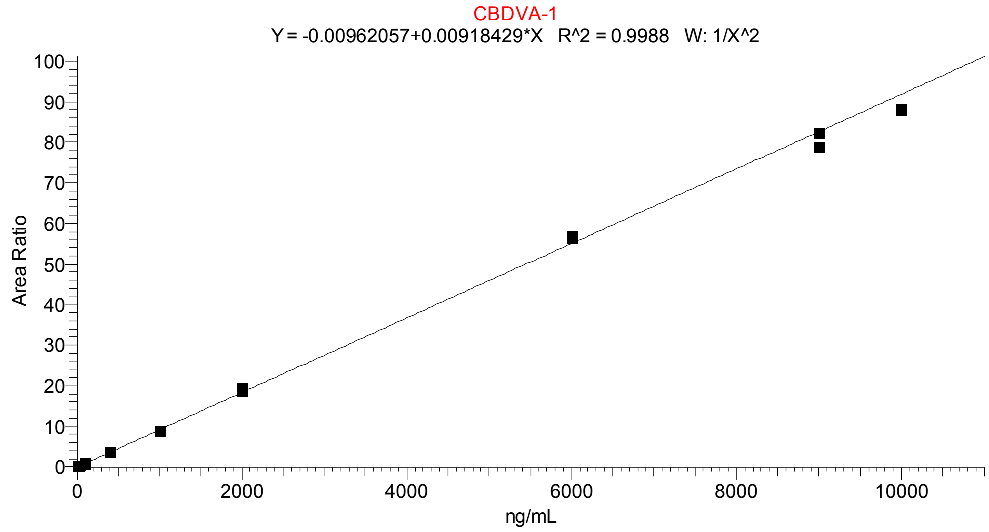

Fig. S14 CBDVA Linear regression, weighted $1/x^2$ (duplicate injection of a calibration curve, beginning and end of batch)

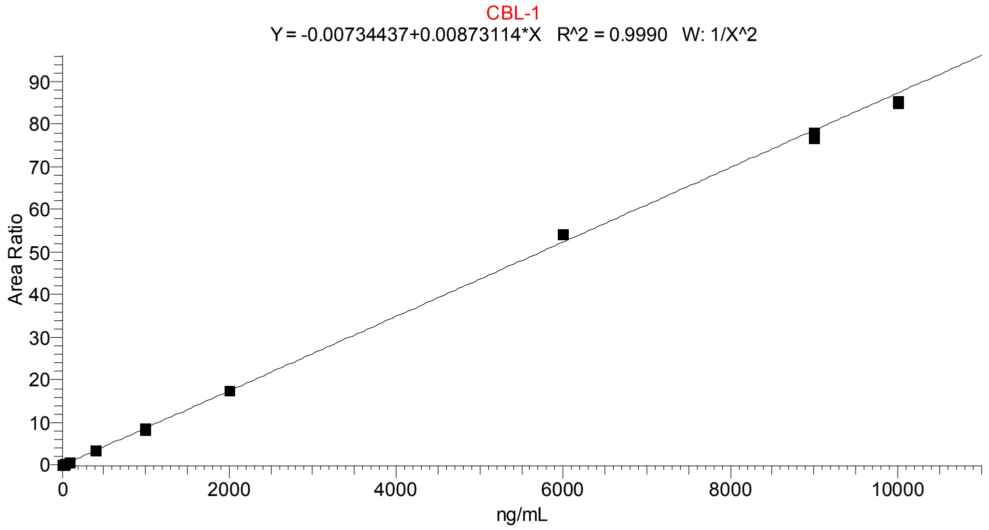

Fig. S15 CBL Linear regression, weighted $1/x^2$ (duplicate injection of a calibration curve, beginning and end of batch)

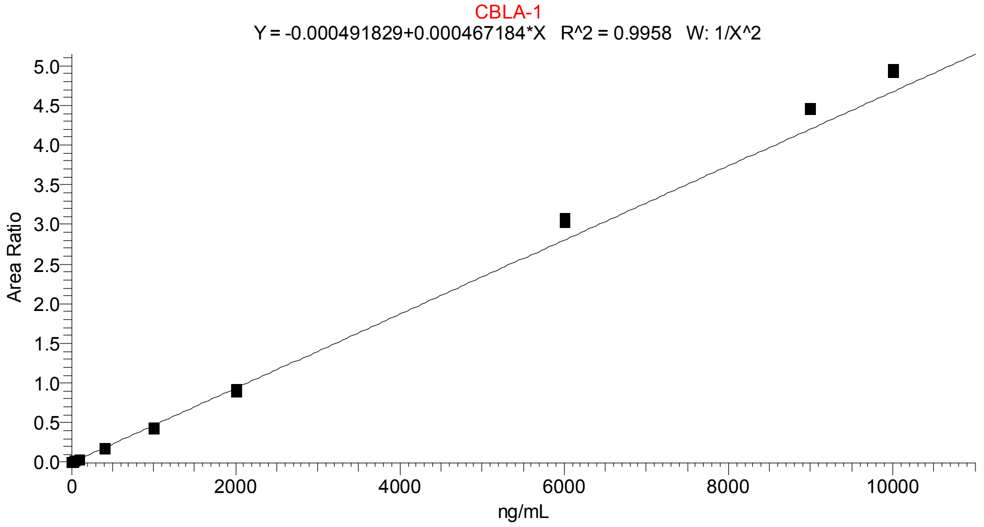

Fig. S16 CBLA Linear regression, weighted $1/x^2$ (duplicate injection of a calibration curve, beginning and end of batch)

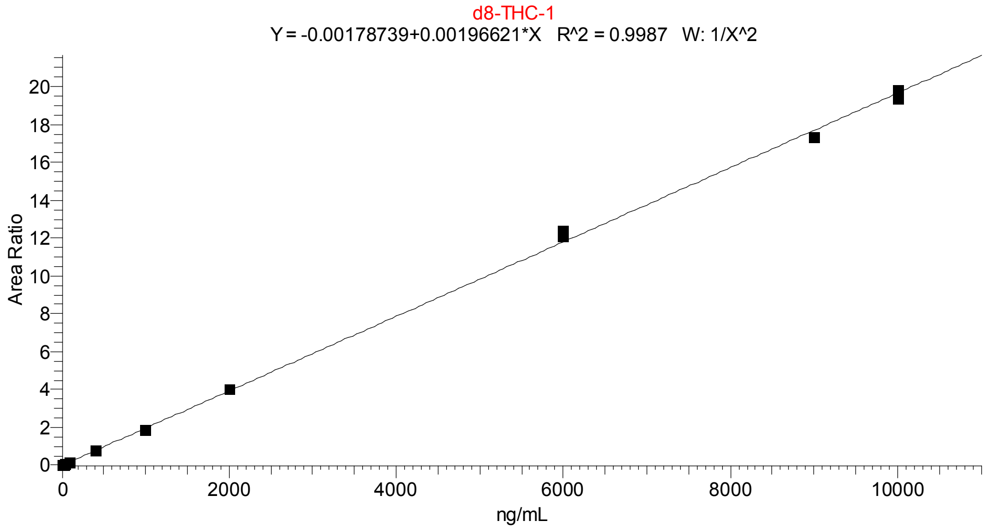

Fig. S17 Δ8-THC Linear regression, weighted $1/x^2$ (duplicate injection of a calibration curve, beginning and end of batch)
